# Characterization of *Ictalurid herpesvirus* 1 Glycoprotein ORF59 and Its Potential Role on Virus Entry into the Host Cells

**DOI:** 10.3390/v13122393

**Published:** 2021-11-29

**Authors:** Shu-Xin Li, Fei Yu, Hong-Xun Chen, Xiao-Dong Zhang, Li-Hui Meng, Kai Hao, Zhe Zhao

**Affiliations:** 1 Department of Marine Biology, College of Oceanography, Hohai University, Nanjing 210098, China; lishuxin@hhu.edu.cn (S.-X.L.); 20190029@hhu.edu.cn (F.Y.); chenhongxun@hhu.edu.cn (H.-X.C.); zhangxiaodong@hhu.edu.cn (X.-D.Z.); menglihui@hhu.edu.cn (L.-H.M.); haokai87@hhu.edu.cn (K.H.); 2 State Key Laboratory of Freshwater Ecology and Biotechnology, Institute of Hydrobiology, Chinese Academy of Sciences, Wuhan 430072, China

**Keywords:** *Ictalurid herpesvirus* 1, glycoprotein ORF59, baculovirus expression, protein blocking, virus replication

## Abstract

The channel catfish virus (CCV, *Ictalurid herpesvirus* 1) has caused sustained economic losses in the fish industry because of its strong infectivity and pathogenicity. Thus, it is necessary to determine the function of viral proteins in the CCV infection process. The present study aimed to characterize CCV glycoprotein ORF59 and explore its impact on virus infection in host cells. Firstly, its exclusive presence in the membrane fraction of the cell lysate and subcellular localization verified that CCV ORF59 is a viral membrane protein expressed at late-stage infection. A protein blocking assay using purified His6 tagged ORF59, expressed in sf9 insect cells using a baculovirus expression system, indicated a dose-dependent inhibitory effect of recombinant ORF59 protein on virus invasion. Knockdown of the *ORF59* using a short hairpin (shRNA) showed that *ORF59* silencing decreased the production of infectious virus particles in channel catfish ovary cells. The results of this study suggest that recombinant ORF59 protein might inhibit CCV entry into the host cells. These findings will promote future studies of the key functions of glycoprotein ORF59 during CCV infection.

## 1. Introduction

The channel catfish (*Ictalurus punctatus*) is an important aquaculture species with high economic value in the commercial markets. Channel catfish virus (CCV, *Ictalurid herpesvirus 1*), a member of viruses of the genus *Ictalurivirus* (family: *Alloherpesviridae*), causes severe hemorrhagic disease in channel catfish. To date, the severity of epizootic channel catfish virus disease (CCVD) has increased significantly [1], which has caused significant economic losses and affected the healthy development of the channel catfish industry. The 134 kilo base-pair (kb) linear double-stranded DNA genome of CCV encodes 79 potential genes, 14 of which are located in terminal repeat regions of the genomic DNA [2]. Similar to all herpesviruses, the CCV DNA genome is packaged within an icosahedral nucleocapsid (capsid diameter of ~100 nm), which is surrounded by a proteinaceous tegument layer and a trilaminar membrane (diameter of 175–200 nm) [3].

Previously, 37 structural proteins have been identified by using liquid chromatography electrospray ionization tandem mass spectrometry (LC/ESI-MS/MS) technology from CCV particles. Based on bioinformatic analysis, these proteins were further grouped as 4 capsid proteins, 5 tegument proteins, 3 envelope proteins, and 25 unclassified proteins [4]. Among the viral structural proteins, the predicted ORF59 protein is encoded by the CCV ORF59 gene, and this hydrophobic membrane protein is considered as the major envelope glycoprotein of CCV ORF59 protein that contains four stretches of hydrophobic residues capable of spanning the membrane [5], and an orientation in which the termini are inside the envelope would position a loop containing three potential N-linked glycosylation sites on the external surface of the virion [6].

Among viral structural proteins of mammalian herpesviruses, the envelope protein is very important for virus invasion and assembly because of the involvement of this protein in host–virus interactions, such as attachment to cellular receptors and fusion with the host cell membrane during viral infection [7]. In the case of alphaherpesviruses, virus attachment to the cell surface is a charge-based or a relatively nonspecific process by which viral envelope glycoproteins (gC and gB etc.) associate with chondroitin/heparan sulfate proteoglycans or several proteins of the host cell [8,9]. As a result, blocking the interaction between viral glycoproteins and these cellular receptors, using either blocking antibodies or soluble glycoproteins, can inhibit herpesvirus entry into host cells. Virus neutralizing antibodies defined in vitro have been proposed to confer protection against herpesvirus infection, and the virion envelope glycoproteins serve as major targets of neutralizing antibodies [10]. For instance, utilizing a panel of HCMV virus-neutralizing gB-specific monoclonal antibodies (mAbs), syncytium formation of the fusogenic gB/VSV-G chimera was significantly inhibited by only a subset of neutralizing mAbs that target antigenic domain 5 (AD-5) of gB [11]. Additional glycoproteins of herpesviruses on the cellular surface can also inhibit viral infection by abrogation of cellular binding.

Viral glycoproteins play important roles in the pathogenesis of herpesvirus infections. However, few data concerning the characterization of CCV glycoproteins are available. Analysis of amino acid sequences and experimental evidence revealed that a mucin-like glycoprotein of CCV is encoded by ORF50 [12]. This protein possesses several N-glycosylation sites and it is secreted into the outside of cells with the help of a cleavable signal sequence during virus infection. Nevertheless, glycoprotein ORF59 of CCV can be detected and recognized by anti-CCV virion serum, which indicated that ORF59 serves as a structural envelope glycoprotein of the CCV virion [3]. Currently, there is no evidence to show that CCV ORF59 shares amino-acid sequence homology with other envelope glycoproteins of mammalian and avian herpesviruses. The present study aimed to confirm that glycoprotein ORF59 is an envelope protein and to explore its impact on CCV infection during in vitro infection.

## 2. Materials and Methods

### 2.1. Cell and Virus

Channel catfish ovary (CCO) [13,14] cells were cultured in Dulbecco’s modified Eagle’s medium (DMEM) medium (Gibco, Big Cabin, OK, USA) supplemented with 10% fetal bovine serum (FBS, Gibco), penicillin (100 IU/mL), and streptomycin (0.1 mg/mL) (Sigma, St. Louis, MO, USA) and maintained at 28 °C. Spodoptera frugiperda cells (Sf9) were grown in suspension and monolayer cultures at 28 °C in serum free medium, SF900 II SFM (Gibco, USA).

CCV (strain VR-665) was kindly provided by Prof. Jun-Fa Yuan, Huazhong Agricultural University, Wuhan, Hubei, China, and propagated in CCO cells containing DMEM supplemented with 10% FBS until a cytopathic effect (CPE) was observed. Subsequently, the virus was harvested and stored at −80 °C for further use. The virus was titrated into 96-well plates, and a TCID50 (median tissue culture infectious dose) analysis was performed according to the Reed–Muench method [15].

### 2.2. Antibodies Preparation

Specific amino acid sequences of CCV ORF39 (457–474 aa, and 1031–1045 aa) and ORF59 (299–316 aa and 75–90 aa) were initially synthesized and selected, then were coupled with KLH carrier protein. New Zealand white rabbits were vaccinated to collect rabbit serum to prepare rabbit anti-ORF39 and 59 polyclonal antibodies (ABclonal, Wuhan, China), as previously reported [16]. The following antibodies were purchased commercially: HRP Goat Anti-Mouse IgG (H + L) (ABclonal, China), Mouse anti His-Tag mAb (ABclonal, China), Mouse anti GFP-Tag mAb (ABclonal, China), GAPDH Rabbit mAb (ABclonal, China), HRP Goat Anti-Mouse IgG (H + L) (Beyotime, Haimen, China), and HRP Goat Anti-Rabbit IgG (H + L) (Beyotime, China).

### 2.3. Plasmid Construction

The coding sequence of the CCV ORF59 protein was from CCV *ORF59* (GenBank accession no. NP_041150.1). Two pairs of primers (eGFP-ORF59F/R; pBHTA-59F/R, Table 1) were designed to clone and express the corresponding CCV ORF59 fragment using template DNA from CCV-infected CCO cells. The amplified PCR products of the appropriate size were separated by agarose gel electrophoresis, purified using the Gel Extraction kit (Omega Bio-Tek, Norcross, GA, USA). Amplified products were cloned into vector pEGFP-N3 (Clontech, Mountain View, CA, USA) or vector pFastBac™ HT A (Invitrogen, Waltham, MA, USA) to construct recombinant plasmids for gene expression.

### 2.4. Design and Synthesis of Short Hairpin RNAs (shRNAs)

The DNA constructs of 53 bp in length targeting the CCV *ORF59*, which could be transcribed into shRNAs in cells, were designed according to Li’s method and web-based criteria (www.ambion.com, accessed on 22 November 2020) and chemically synthesized [17,18]. The 5′ ends of the synthesized oligonucleotides were phosphorylated to facilitate the ligation reaction. A *BamH I* restriction site overhang was added at the ends of the sense shRNA template strands. At the ends of the antisense strands, a *BasI* restriction site overhang was added for the efficient directional cloning of the DNA constructs into vector pGPU6-GFP-Neo (GenePharma, Shanghai, China), yielding plasmids shRNA59-158, shRNA59-257, shRNA59-422, and shRNA59-948. The negative control sequence, shNc, was provided by the pGPU6-GFP-Neo vector kit.

### 2.5. Plasmid Transfection

Plasmids were transfected into CCO cells using Lipofectamine 2000 (Invitrogen, USA) as described in manufacturer’s instructions [19]. Firstly, CCO cells were seeded in a 6-well plate (Corning, New York, NY, USA) at 2 × 10^5^ cells per well in DMEM supplemented with 10% FBS without antibiotics. Cells were incubated at 28 °C and grown to about 80% confluency. Using the optimal transfection conditions, 2.5 μg of plasmids DNA diluted in 250 μL of OptiMEM medium (Gibco, USA) were mixed with 10 μL of Lipofectamine 2000 diluted in 250 μL OptiMEM and then incubated at room temperature for 20 min [20]. The DNA complexes were added to cells and incubated for 6 h at 28 °C. The cells were then washed and infected with 1000 TCID50/mL CCV.

### 2.6. CCV DNA Synthesis Inhibition

CCO cells were infected with 1000 TCID50/mL CCV for 1 h or mock-infected in the presence (or absence) of cytosine β-d-arabinofuranoside (Ara-C; 100 μg/mL) [21]. Cell samples were collected at 4, 8, 12, 16, and 24 hpi. Total RNA was extracted using Trizol (Invitrogen, USA) following the manufacturer’s instructions. Synthesis of cDNA was accomplished according to the manufacturer’s instructions using a PrimeScript RT reagent Kit with gDNA Eraser (Takara, Kusatsu, Japan). The primers used for PCR in this study are listed in Table 1. Dimethyl sulfoxide (DMSO) served as negative control.

### 2.7. Fluorescence Observation

CCO cells were inoculated on a microscopic coverslip in 12-well plates grown to 90% confluence. Then, 2.5 μg of each recombinant plasmid were transfected into CCO cells using the lipofectamine 2000 (Invitrogen, USA) reagent (according to the manufacturer’s instruction). At 24 h post-transfection (hpt), cells were fixed with 4% paraformaldehyde (PFA) for 30 min, permeabilized with 0.1% Triton X-100 for 15 min, and stained with Hoechst 33342 (Sigma, USA) for 15 min. The cells were observed under a LSM 900 with Nikon Eclipse Ti (objective 1000×) (Nikon, Tokyo, Japan).

### 2.8. Quantitative Real-Time Reverse Transcription PCR

Total RNA was extracted using Trizol (Invitrogen, USA) following the manufacturer’s instructions. RNA concentrations were quantified using a Mettler UV5 NANO (Mettler, Zurich, Switzerland). The purity of the extracted RNA was determined using an A260 nm/A280 nm ratio with absorbance at 260 and 280 nm between 1.8 and 2.0, respectively. Synthesis of cDNA was accomplished according to the manufacturer’s instructions using a PrimeScript RT reagent Kit with gDNA Eraser (Takara, Japan). The resultant cDNA was quantified using TB Green Premix Ex Taq (Takara, Japan) in a LightCycler96 Real-Time PCR Detection System (Roche, Basel, Switzerland). The quantitative real-time PCR (qPCR) protocol was as follows: 1 cycle of 95 °C for 30 s, 40 cycles of 95 °C for 5 s, and 60 °C for 30 s. The primers used for qRT-PCR in this study are listed in Table 1. Each individual sample was run in triplicate wells and fold changes of mRNA expression were calculated using the 2^−^^△△Ct^ method [22].

### 2.9. Cell Plasma Membrane Protein Isolation

Membrane proteins were extracted from 5 × 10^6^ cells using a Minute plasma membrane protein isolation and cell fractionation kit (Beyotime, China) following the manufacturer’s instructions. The pellets containing plasma membrane proteins and nonmembrane proteins were collected to analyze ORF59 expression using Western blotting. Enhanced green fluorescent protein (eGFP) was used as plasma membrane loading control.

### 2.10. Virus Titration

To test whether plasmid-transcribed shRNAs could inhibit the production of infectious CCV, viral supernatants of the various treatment groups collected at 24 hpi were freeze–thawed twice, centrifuged, and diluted from 10^−1^ to 10^−9^ in DMEM for TCID50/mL determination. Viral titration was performed in CCO cells cultured in 96-well plates (Corning, USA) with eight repetitions per dilution. Viral titers were calculated using the method described by Reed–Muench.

### 2.11. Baculovirus Expression of ORF59 Protein

The pFastBac-HT-ORF59 plasmid was transformed into DH10Bac *E. coli* (Invitrogen, USA) to generate a recombinant bacmid. Positive colonies were selected on Luria–Bertani agar (LB) plates containing tetracycline, kanamycin, and gentamicin as selective antibiotics. X-gal (5-bromo-4-chloro-3-indolyl-β-D-galactosidase) and IPTG (isopropylthio-P-galactosidase) were used for color selection. The PureLink™ HiPure Plasmid Filter Midiprep kit (Invitrogen, USA) was used to achieve a recombinant bacmid. Then, 2 μg of the purified recombinant bacmid DNA was transfected into Sf9 cells (1–2 × 10^6^) to generate a recombinant baculovirus using Cellfectin^®^ II Reagent (Thermo, Waltham, MA, USA) according to the manufacturer’s instructions. At 72 h after transfection, the Sf9 cells and medium containing recombinant baculovirus (baculovirus-His6-ORF59) were harvested and used to detect ORF59 expression and amplify the next generation viruses (baculoviral stock). The baculoviral stock was used to infect Sf9 cells to express the His6-ORF59 recombinant protein, which was purified using Ni-NTA His•Bind^®^ Resins protocols (Millipore, Burlington, MA, USA). The concentrations of the proteins used in this study, including purified His6-ORF59, were measured using an Enhanced BAC Protein Assay kit (Beyotime, China).

### 2.12. Western Blotting Assay

Protein samples were denatured by boiling before electrophoresis in a 10% SDS polyacrylamide gel. Proteins were visualized by staining with either Coomassie blue (Beyotime) or transferred to polyvinylidene fluoride (PVDF) membranes (Millipore, USA). The PVDF membranes were blocked for 2 h at room temperature with 5% skim milk (Sangon, Shanghai, China) in PBST (0.05% Tween in 0.01 M phosphate-buffered saline (PBS)). Blocked membranes were then incubated with primary mouse anti His-Tag mAbs (ABclonal, China) diluted in 2% skim milk in PBST for 2 h at 25 °C and washed three times with PBST. Membranes were then incubated with horseradish peroxidase (HRP)-Goat Anti-Mouse IgG (H + L) (ABclonal, China) secondary antibodies in 2% skim milk in PBST for 2 h at 25 °C and were washed extensively. The signals were detected using enhanced chemiluminescence (ECL) reagents (Thermo, USA).

### 2.13. Protein Blocking Assay

CCO cells were incubated with different concentrations of ORF59 protein at 28 °C for 2 h. The cells were infected with 1000 TCID50/mL CCV for 1 h. Cell samples were collected and the virus genome was measured using qPCR at 12, 16, and 24 hpi. Bovine serum albumin (BSA) served as negative control. The virus plaques were observed by microscope (Axio Vert A1, 10×). The ultrastructural morphology of the virions within infected cells was examined using transmission electron microscopy (TEM) as described previously [23,24].

### 2.14. Statistical Analysis

Data are presented as means ± standard error of mean (SEM). qRT-PCR and qPCR data were analyzed using one-way analysis of variance followed by LSD test (least significant difference test) using SPSS 20. 0 (IBM, Armonk, NY, USA). Significance was set at *p* value < 0.05.

## 3. Results

### 3.1. CCV ORF59 Is a Late Expression Gene during CCV Infection

**Figure 1:**
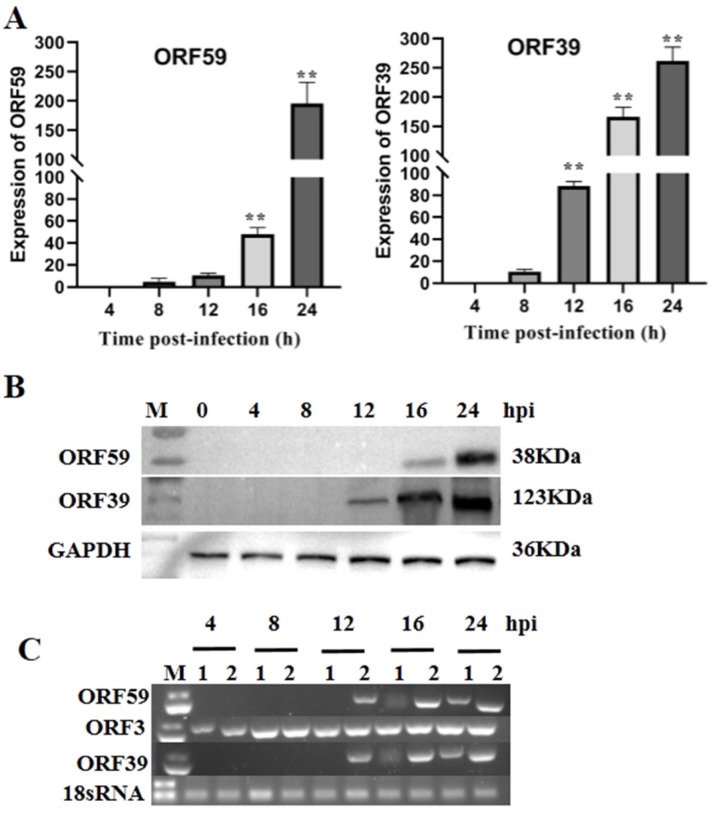
Kinetics of ORF59 expression in infected cells. (**A**) Relative abundance of CCV *ORF59* and *ORF39* mRNAs. CCO cells were infected with 1000 TCID50/mL CCV and samples were collected at 4, 8, 12, 16, and 24 hpi. The relative fold change was measured using qRT-PCR normalized against 18S rRNA, and data were compared with the 4 h group. Values represent mean ± SD (n > 3) for experiments performed in triplicate. ** *p* < 0.01. (**B**) CCV ORF59 and ORF39 proteins were analyzed by Western blotting. GAPDH served as an internal reference protein, and protein size markers are indicated (lane M). CCV, channel catfish virus; CCO, channel catfish ovary; hpi, hours post-infection; qRT-PCR, quantitative real-time reverse transcription PCR; GAPDH, glyceraldehyde-3-phosphate dehydrogenase. (**C**) PCR analysis of CCV early and late genes. Total RNA was isolated from CCV-infected (4, 8, 12, 16, 24 hpi.) and mock-infected CCO cells in the presence or absence of cytosine β-D-arabinofuranoside (Ara-C; 100 μg/mL). The PCR reaction was analyzed on a 1.0% agarose gel. Lane: 1, Ara-C; Lane 2, DMSO. 18sRNA served as an internal reference gene.

The expression of CCV genes during infection of CCO cells can be classified into three main phases, including immediate–early (IE), early, and late phases, similar to those of other herpesviruses [25]. The temporal expression of CCV *ORF59* was characterized during CCV infection over a 24 h time course using qRT-PCR and Western blotting (Figure 1A). qRT-PCR results showed that the abundance of *ORF59* mRNA increased at 12 h post-infection (hpi) and reached the higher level at 24 hpi, indicating that the *ORF59* is a CCV late gene. This conclusion was supported by Western blot analysis on ORF59 expression in the protein level (Figure 1B). Similarly, the temporal expression of ORF39, a known late gene of CCV used as control, exhibited a consistent pattern with that of ORF59 in both mRNA and protein levels (Figure 1). Obviously, the CCV ORF3 was detected at 4 hpi, while CCV ORF59 and ORF39 were detect at 12 hpi. Moreover, we found that transcriptions of ORF59 and ORF39 were inhibited by Ara-C, a DNA synthesis inhibitor (Figure 1C). These results confirmed that CCV *ORF59* belongs to the late expression class of CCV genes [21].

### 3.2. CCV ORF59 Is a Membrane Associated Protein

**Figure 2:**
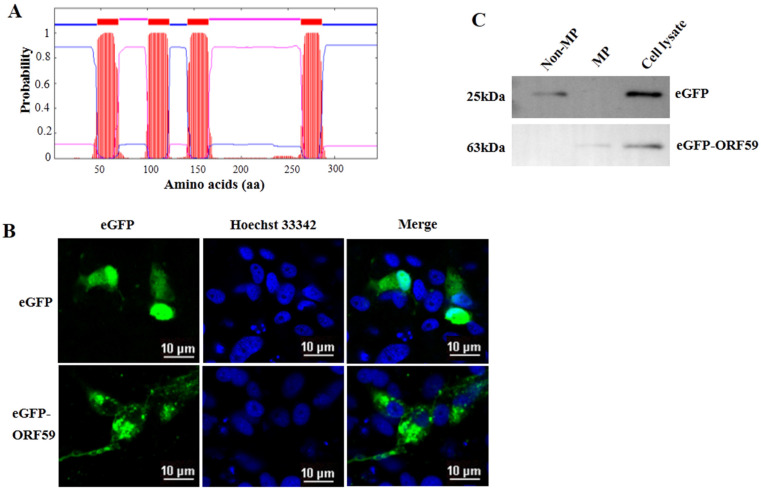
ORF59 is a membrane protein in CCO cells. (**A**) Prediction of transmembrane regions of the ORF59 protein. The ORF59 amino acid sequence was analyzed using TMHMM software. The plot shows the probability of transmembrane helices, and the positions of amino acids are indicated. (**B**) Intracellular localization of ORF59 detected by expression of an eGFP-ORF59 fusion protein. The plasmids were transfected into CCO cells. Representative images were obtained under fluorescence channels. Bar = 10 μm. (**C**) Analysis of ORF59 expression using Western blotting. Membrane proteins (MP) and nonmembrane proteins (Non-MP) were extracted from CCO cells transfected with pEGFP-ORF59. eGFP and the ORF59 fusion protein were probed using specific anti-eGFP antibodies and samples of cell lysates served as controls. The predicted molecular weights are shown on the left. CCO, channel catfish ovary; eGFP, enhanced green fluorescent protein.

Four transmembrane domains (TM domains) were predicted in the middle region of ORF59 protein (47–69 aa, 101–123 aa, 143–165 aa, 264–286 aa) (Figure 2A). Moreover, the conserved TM domains are also low complexity regions, consisting of leucine (L), isoleucine (I), and other hydrophobic amino acids. To further confirm CCV ORF59 as a membrane-associated protein, the plasmid eGFP-ORF59 was constructed to express fusion ORF59 with an N-terminal eGFP tag (Figure 2B). Subcellular localization indicated that the ORF59 fusion protein localized in the cytoplasm with a punctate distribution in CCO cells. Subcellular fractionation was assayed in ORF59-expressing CCO cells and subjected to Western blotting analysis (Figure 2C). The results showed that the ORF59 fusion protein was exclusively present in the membrane fraction of the cell lysate, suggesting that the ORF59 is a membrane-associated protein. Taken together, these results indicated that the glycoprotein ORF59 function as a viral envelope protein of CCV.

### 3.3. Recombinant ORF59 Protein Blocks CCV Infection by Abrogation of Cellular Binding

**Figure 3:**
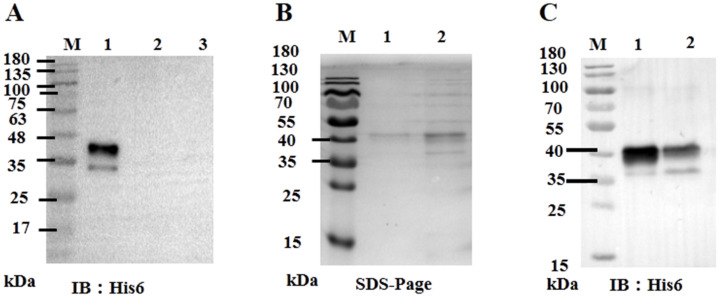
Expression of ORF59 protein in Sf9 cells. (**A**) Western blotting analysis of ORF59 in recombinant baculovirus-infected Sf9 cells. Total cell lysates collected at 72 hpi with recombinant baculovirus were analyzed using Western blotting, and the anti-His6 tag antibody was used to probe the His6-tagged fusion protein ORF59. Lane: 1, Lysates from sf9 cells infected with recombinant baculovirus; Lane: 2, Lysates from sf9 cells infected with control baculovirus; Lane: 3, Sf9 cell lysates. (**B**) SDS-PAGE of expressed and purified tagged fusion protein ORF59. Lanes: 1 and 2, purified fusion protein. (**C**) Western blotting analysis of the expressed and purified tagged ORF59 fusion protein. Lane: 1, purified fusion protein; Lane 2, nonpurified fusion protein. Protein size markers are indicated (lane M). SDS-PAGE, sodium dodecyl sulfate polyacrylamide gel electrophoresis; hpi, hours post-infection.

Given that ORF59 serves as the major envelope glycoprotein of CCV and its potential key function in virus invasion, we further investigated whether exogenous ORF59 protein could block virus-cell binding. Firstly, the recombinant bacmid pBHTA 59 DNA was transfected into insect SF9 cells to obtain the recombinant virus and express the ORF59 protein (Figure S1 and Figure 3A). Soluble ORF59 protein (0.63 mg/mL) was purified from SF9 cells infected with recombinant virus (Figure 3B,C).

**Figure 4:**
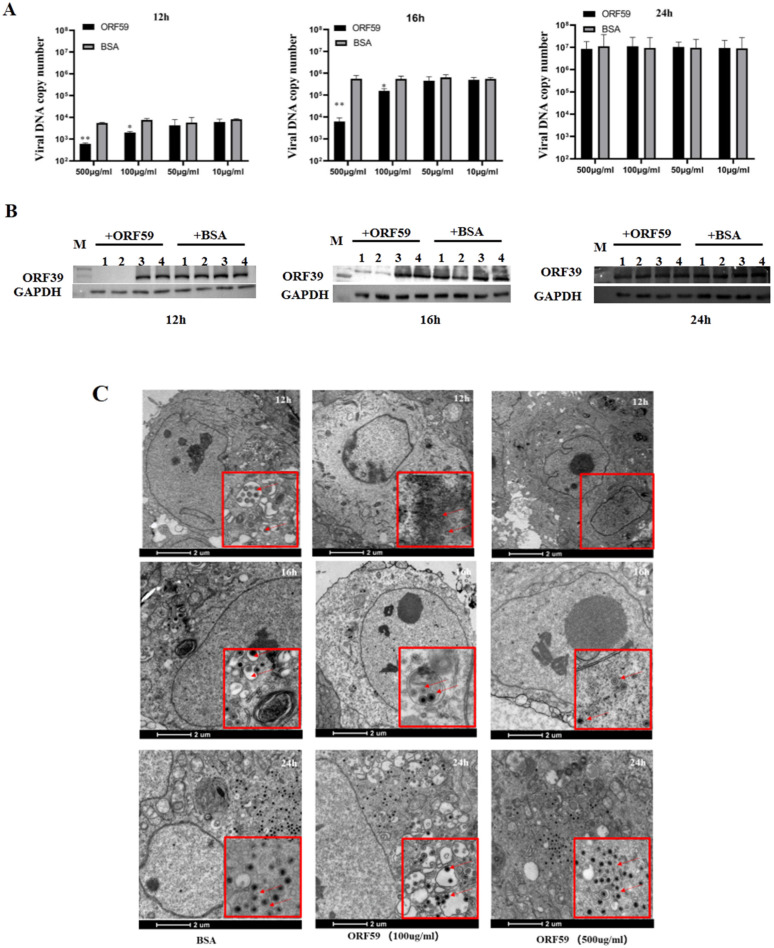
Recombinant ORF59 protein blocks CCV infection. (**A**) CCO cells were pre-incubated with different concentrations of ORF59 proteins at 28 °C for 2 h, and then infected with 1000 TCID50/mL CCV. Cell samples were collected and the virus genome was measured using qPCR at 12, 16, and 24 hpi. BSA protein served as a negative control. Values represent mean ± SD (n > 3) for experiments performed in triplicate. * *p* < 0.05, ** *p* < 0.01. (**B**) The protein samples in A were analyzed by Western blotting at 12, 16, and 24 hpi. The viral major capsid protein ORF39 was probed using specific antibody, and GAPDH served as an internal reference protein. Lane: 1, CCO cells incubated with 500 μg/mL concentrations of ORF59 proteins; Lane: 2, 100 μg/mL; Lane: 3, 50 μg/mL; Lane: 4, 10 μg/mL. (**C**) The ultrastructural morphology of virions within infected cells examined using transmission electron microscopy. CCO cells were pre-incubated with different concentrations of ORF59 proteins (500 and 100 μg/mL) and BSA (500 μg/mL) served as a negative control. Arrows show viral particles. Bar = 2 μm. CCV, channel catfish virus; CCO, channel catfish ovary; hpi, hours post-infection; qPCR, quantitative real-time PCR; BSA, bovine serum albumin; GAPDH, glyceraldehyde-3-phosphate dehydrogenase.

In protein blocking assay, the CCO cells were incubated with different concentrations of soluble ORF59 protein before infection with CCV. Then, the copy number of CCV genomic DNA and the expression level of the viral capsid protein in the CCO cells were measured to reflect the viral infection efficiency (Figure 4A,B). The results showed that pre-incubation of CCO cells with ORF59 protein at 500 μg/mL or 100 μg/mL significantly reduced the viral genomic copy number and protein expression level of ORF39 at 12 and 16 h. Nevertheless, no significant difference at 24 h between all ORF59 blocking groups and control groups was observed. The results were confirmed by TEM examination of the ultrastructural characteristics of infected CCO cells that were pre-treated with 100 μg/mL and 500 μg/mL soluble ORF59 protein (Figure 4C). The data showed that there were fewer virus particles in the ORF59 protein-treated group at 12 and 16 h, while many unenveloped particles were seen in the cytoplasm of the control group cells. Likewise, there were no significant differences at the late stage of virus infection (24 h). We found that there were smaller viral plaques in the ORF59 protein-treated group at 12 and 16 h compared to the control group cells (Figure S2). Together with these results, we concluded that the protein blocking with recombinant ORF59 affected the CCV infection, suggesting that ORF59 protein might block a receptor on the cell surface to delay the first step of infection.

### 3.4. ORF59 Knockdown Reduces Production of Infectious Virus Particles in CCO Cells

**Figure 5:**
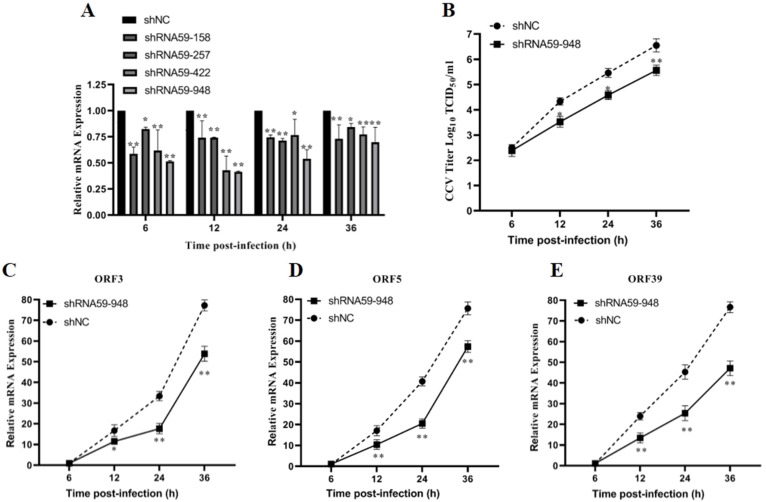
ORF59 silencing affects production of CCV infectious virus particles in CCO cells. (**A**) Relative gene expression of CCO cells after transfecting different shRNA plasmids. RT-PCR was used to determine the genome copy number at 6, 12, 24, and 36 hpi after transfecting plasmid shRNA59-158, shRNA59-257, shRNA59-422, and shRNA59-948. The data were compared with shNC group. (**B**) Measurement of CCV virus titer after ORF59 silencing. The titers of CCV in the CCO supernatant were calculated by the method described by Reed–Muench at the indicated time. (**C**–**E**) The progeny viruses within the cell supernatants at 36 hpi (shRNA59-948 and shRNA-NC) were collected to infect CCO cells, and viral gene expression (ORF3, ORF5, and ORF39 genes) of progeny viruses was determined using qRT-PCR at 6, 12, 24, and 36 hpi. The relative fold change was measured using qRT-PCR normalized against 18S rRNA, and data were compared with the 6 hpi group. CCV, channel catfish virus; CCO, channel catfish ovary; qPCR, quantitative real-time PCR; hpi, hours post-infection; qRT-PCR, quantitative real-time reverse transcription PCR. Values represent mean ± SD (n > 3) for experiments performed in triplicate. * *p* < 0.05, ** *p* < 0.01.

The data provided above implied that CCV ORF59, as a viral membrane glycoprotein, plays a key function in the attachment of infectious virus particles. To investigate the effect of ORF59 expression on the production of infectious virus particles in CCO cells, we knocked down the ORF59 mRNA in CCV-infected CCO cells by RNA interference. Firstly, the knockdown efficiency of 4 shRNA (shRNA59-158, shRNA59-257, shRNA59-422, and shRNA59-948) was evaluated using qRT-PCR analysis, and showed that shRNA-948 exhibits the highest knockdown efficiency against ORF59 mRNA (Figure 5A). Then, virus titers of cell supernatants were measured at different time points in the CCO cells infected with CCV that pre-transfected with the shRNA59-948 plasmid. The result revealed that ORF59 silencing reduced the CCV titers at 12, 24, and 36 hpi, respectively (Figure 5B). Meanwhile, after transfecting with plasmid shRNA59-948, the progeny viruses within the cell supernatants at 36 hpi (shRNA59-948 and shRNA-NC) were collected to infect CCO cells, and viral gene expression (ORF3, ORF5, and ORF39 genes) was further determined using qRT-PCR. The results showed that mRNA levels of three CCV genes (ORF3, ORF5, and ORF39) were obviously decreased in infected CCO cell at 12, 24, and 36 hpi, compared with those of the shRNA-NC group (Figure 5C–E). Moreover, the qPCR results showed that the shRNA59-948 group significantly reduced the viral genomic copy number at 12 and 16 hpi (Figure S3). Overall, these findings suggested that ORF59 silencing could reduce the production of infectious virus particles in CCO cells.

## 4. Discussion

Late genes of herpesviruses, mostly encoding viral structural proteins, are generally expressed in the late stage of infection and regulated by early or IE gene products [26]. CCV genes, including ORFs 4, 7, 10–13, and 39 were classified as the late genes [21,27,28]. In this study, CCV ORF59 was verified as a potential viral late-stage expression gene and was expressed in the cytoplasm. Although ORF59 was characterized as the major envelope glycoprotein of the CCV virion, there is no evidence to prove the characteristics of cell membrane positioning in fish cells. Bioinformatics and the membrane fraction analysis revealed that glycoprotein ORF59 is a membrane-associated protein [28]. Similar to the members of subfamily *Alphaherpesvirinae*, the CCV virion is composed of multiple architectural features: envelope, tegument, capsid, and the viral genome, and the viral structural envelope glycoproteins are the key composition of the virion [29]. Taken together, these observations indicated that CCV glycoprotein ORF59 might be relevant to virion assembly at the late-stage of infection to form the integral viral particles.

In addition, the glycoproteins of enveloped viruses fulfill three major functions to enable virus entry into target cells: the attachment of virions to cells, a step that partly determines the type of cells that the virus targets, i.e., the viral tropism; the triggering of fusion, i.e., the activation of the fusion machinery; and the execution of fusion [30]. However, few data are available concerning the function of the CCV glycoproteins. Our study not only characterized CCV glycoprotein ORF59, but also preliminarily explored its function. Glycoprotein ORF59, as the major envelope glycoprotein of CCV virions, forms the complete structure of CCV particles and is involved in inhibiting the early events taking place between CCV and host cells [3]. We therefore speculated that CCV ORF59 might inextricably link with host cell receptor proteins and plays a key role in the process of virus invasion. Virus entry into host cells is a complex process involving interactions between an array of viral glycoproteins with multiple host cell surface receptors [31]. Blocking herpesvirus glycoproteins or their interaction with receptors has the potential to inhibit viral entry into the host cell and cell-to-cell spread [32,33]. In this study, recombinant ORF59 protein blocking reduced viral genome replication and protein expression, and the TEM observation also showed that infected CCO cells pre-treated with 100 and 500 μg/mL soluble ORF59 protein has less enveloped virus particles than control group cells at 12 and 16 h. These results suggested that the receptor on the surface of the cell might have been blocked, which reveals that glycoprotein ORF59 is a potential protein mediating CCV attachment to host cells.

Herpesviruses assemble capsids and package genomes into those capsids in the nucleus to form de-enveloped capsids. At the late-stage of infection, the de-enveloped capsids must undergo re-envelopment in the cytoplasm to generate infectious viruses [34,35]. Here, ORF59 silencing reduced the CCV titers and mRNA levels of the viral genes (ORF3, ORF5, and ORF39) of the virus progeny in infected CCO cells. Knockdown of ORF59 using a short hairpin (shRNA) reduced the production of CCV infectious virus particles in host cells, which might be caused by a negative effect on the structural integrity of the virus progeny, suggesting that glycoprotein ORF59 is essential for CCV optimal infection [5,28].

CCV, a member of the family *Alloherpesviridae*, which provokes disease and can devastate a cultured channel catfish population, has damaged the aquaculture industry [36]. In recent years, there are increasingly more disturbing reports of channel catfish virus infection in farmed channel catfish. Although CCVD is a well-known problem, reports on the CCV are quite limited. Therefore, it is essential for us to develop new antiviral strategies to control channel catfish virus disease. Our previous work reported that acyclovir inhibits channel catfish virus replication and protects channel catfish ovary cells from apoptosis, which showed that this antivirus drug is a promising agent against CCV in the aquaculture industry [1]. Otherwise, the inhibition of the first step of CCV infection, the entry into host cells, is an alternative anti-CCV strategy. In this study, ORF59 silencing reduced the production of CCV infectious virus particles in host cells, and ORF59 protein blocking also had an inhibitory effect on virus adsorption. Similarly, a partial deletion of the herpes simplex virus UL20 gene which encodes an envelope protein was reported to form weakly syncytial viral plaques in cells and severe defects in cytoplasmic virion envelopment, implying that UL20 function as an antivirus target during HSV infection [37]. The findings of this study also showed that ORF59 might be a potential antivirus target gene for channel catfish virus disease.

For herpesvirus entry, it is usual that a collective of glycoproteins is involved in attachment to the cell surface. After that, specific interactions take place between viral glycoproteins and host cell receptors, and then molecular interactions and triggers occur, ultimately leading to viral envelope fusion with the host cell membrane [38]. Herpesvirus infecting different type cells requires a multimeric complex of glycoproteins gD, gB, gH/gL and other proteins such as pUL128, pUL130, and pUL131A [32,39]. Specifically, HSV attachment and entry into cells require multiple interactions between four essential virion envelope glycoproteins (gD, gB, gH, and gL) and a cellular receptor (nectin-1 or herpesvirus entry mediator (HVEM)) [40]. Nevertheless, results of the present study suggested that glycoprotein ORF59 of CCV is involved in the attachment of virions to cells and this protein might be one of the subunits of multimeric complex of CCV attachment/entry. Further research on the unknown molecules that interact with this glycoprotein in fish cells is required, and the other function(s) of glycoprotein ORF59 during CCV infection should be explored.

## 5. Conclusions

In summary, this study provided evidence that the CCV ORF59 protein is a membrane protein expressed at the late-stage of the virus infection cycle. We also demonstrated that ORF59 protein blocking had an inhibitory effect on virus adsorption, and ORF59 silencing inhibited the production of infectious CCV particles in CCO cells. This evidence indicated that ORF59 protein has a key role in CCV infection, and implies that CCV ORF59 is a targeted gene to inhibit virus replication. Our findings will promote future studies about the function of glycoprotein ORF59 in the process of CCV infection and have significant implications for understanding CCV pathogenesis, which will permit the development of new therapies.

## Figures and Tables

**Table 1:** Primers used in this study.

Primer	Sequence (5′–3′)	Purpose
ORF59-F	AGGCGTATCACCAACTCACC	RT-PCR and PCR
ORF59-R	ACCGAACTGGTGAGGATCAG	RT-PCR and PCR
ORF3-F	GATGAGGGCGACGACACTAT	PCR
ORF-R	AGTCCCAGTCGGAAGTCTCA	PCR
ORF39-F	GAAGATAGCCCGTCTCACCG	PCR
ORF39-R	ATCTCGATCAGCATCTGGCG	PCR
18sRNA-F	CGCCCCGCCCAACTCGCCTGAATA	RT-PCR
18sRNA-R	CGAATGCCCCCGCCGTCCCTCTTA	RT-PCR
eGFP-ORF59F	CTCAAG CTTACCATG GTCGGCAAAGGTCTCC	ORF amplification
eGFP-ORF59R	CCGGGATCCCGCCCGGGCAGGTGTGTAGT	ORF amplification
shRNA59-158F	CACCAACTCACCAAGCTACAAG	RT-PCR
shRNA59-158R	CCTACCAGGTCTATCACCGAAC	RT-PCR
shRNA59-257F	CGGTGATAGACCTGGTAGGG	RT-PCR
shRNA59-257R	GCGACGAAAACGATCATCAG	RT-PCR
shRNA59-422F	ACCTCTTTCGGGTTCGATGT	RT-PCR
shRNA59-422R	TCAGGATACTGAACACCGTGA	RT-PCR
shRNA59-948F	GGTTGGGGACAATAATCGAA	RT-PCR
ahRNA59-948R	TTCTCATACCGGGAATGGTG	RT-PCR
cVRT-F	GAAGATAGCCCGTCTCACCG	qPCR
cVRT-R	ATCTCGATCAGCATCTGGCG	qPCR
shORF3-F	CTGGAATCCTCCTCCTCCTT	qRT-PCR
shORF3-R	GTCGGAGACGGGAGAGTACA	qRT-PCR
shORF5-F	CCGTCTTCGTGTACCTGGAG	qRT-PCR
shORF5-R	CCACGCCTCGTATCTTTCG	qRT-PCR
shORF39-F	GGGTCTCATCTTTGCCGATA	qRT-PCR
shORF39-R	AGTTTGAGCGAGAACCCGTA	qRT-PCR
pBHTA-59F	ttttcagggcgccatggatccGATGGTCGGCAAAGGTCTCCC	ORF amplification
pBHTA-59R	tcgacgtaggcctttgaattcTCACGCCCGGGCAGGTGT	ORF amplification
pUC/M13F	CCCAGTCACGACGTTGTAAAACG	PCR
pUC/M13R	AGCGGATAACAATTTCACACAGG	PCR

## Data Availability

Not applicable.

## Supplementary Materials

The following are available online at https://www.mdpi.com/article/10.3390/v13122393/s1, Figure S1: Identification of recombinant Bacmid-ORF59 by PCR. Figure S2: Observation of virus plaque. Figure S3: Measurement of the copy number of CCV genomic DNA after ORF59 silencing.
