# Supplementary material for: Characterization of Ictalurid herpesvirus 1 Glycoprotein ORF59 and Its Potential Role on Virus Entry into the Host Cells
Source: Viruses. 2021 Nov 29;13(12):2393. doi: 10.3390/v13122393 (PMC8709185; doi:10.3390/v13122393)
Supplement: Supplementary file 1 [file viruses-13-02393-s001.zip › viruses-1453047-supplementary.pdf]

Supplementary Materials:

Characterization of *Ictalurid herpesvirus 1* glycoprotein ORF59 and its potential role on virus entry into the host cells

Shu-xin Li [1 †], Fei Yu [1 †], Hong-Xun Chen [1], Xiao-Dong Zhang [1], Li-Hui Meng[1,2], Kai Hao [1] and Zhe Zhao [1,*]

[1] Department of Marine Biology, College of Oceanography, Hohai University, Nanjing 210098, P.R. China; lishuxin@hhu.edu.cn (S.-X.L.); 20190029@hhu.edu.cn (F.Y.); chenhongxun@hhu.edu.cn (H.-X.C.); zhangxiaodong@hhu.edu.cn (X.-D.Z.); menglihui@hhu.edu.cn (L.-H.M.); haokai87@hhu.edu.cn (K.H.)

[2] State Key Laboratory of Freshwater Ecology and Biotechnology, Institute of Hydrobiology, Chinese Academy of Sciences, Wuhan 430072, P.R. China;

* Correspondence: zhezhao@hhu.edu.cn; Tel.: (86)025-83787653

† These authors contributed equally to this study.

Supplementary Figure. S1. Identification of recombinant Bacmid-ORF59 by PCR.

Supplementary Figure. S2. Observation of Virus plaque.

Supplementary Figure. S3. Measurement of the copy number of CCV genomic DNA after ORF59 silencing.

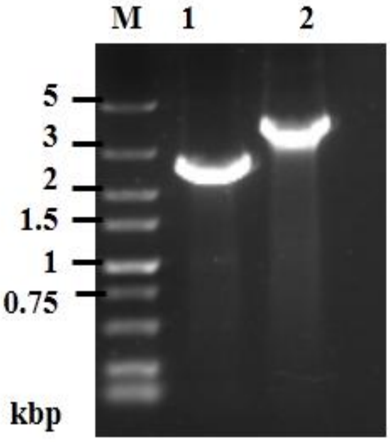

Figure. S1 Identification of recombinant Bacmid-ORF59 by PCR. The pFastBac-HT-ORF59 plasmid was transformed into DH10Bac E. coli to generate a recombinant bacmid, and the bacmid DNA isolated from white and blue colonies was amplified using pUC/M13 primers. The PCR reaction was analyzed on a 1.0% agarose gel. Lanes: 1, pFastBac-HT DNA; Lanes: 2, Bacmid DNA containing the *ORF59*.

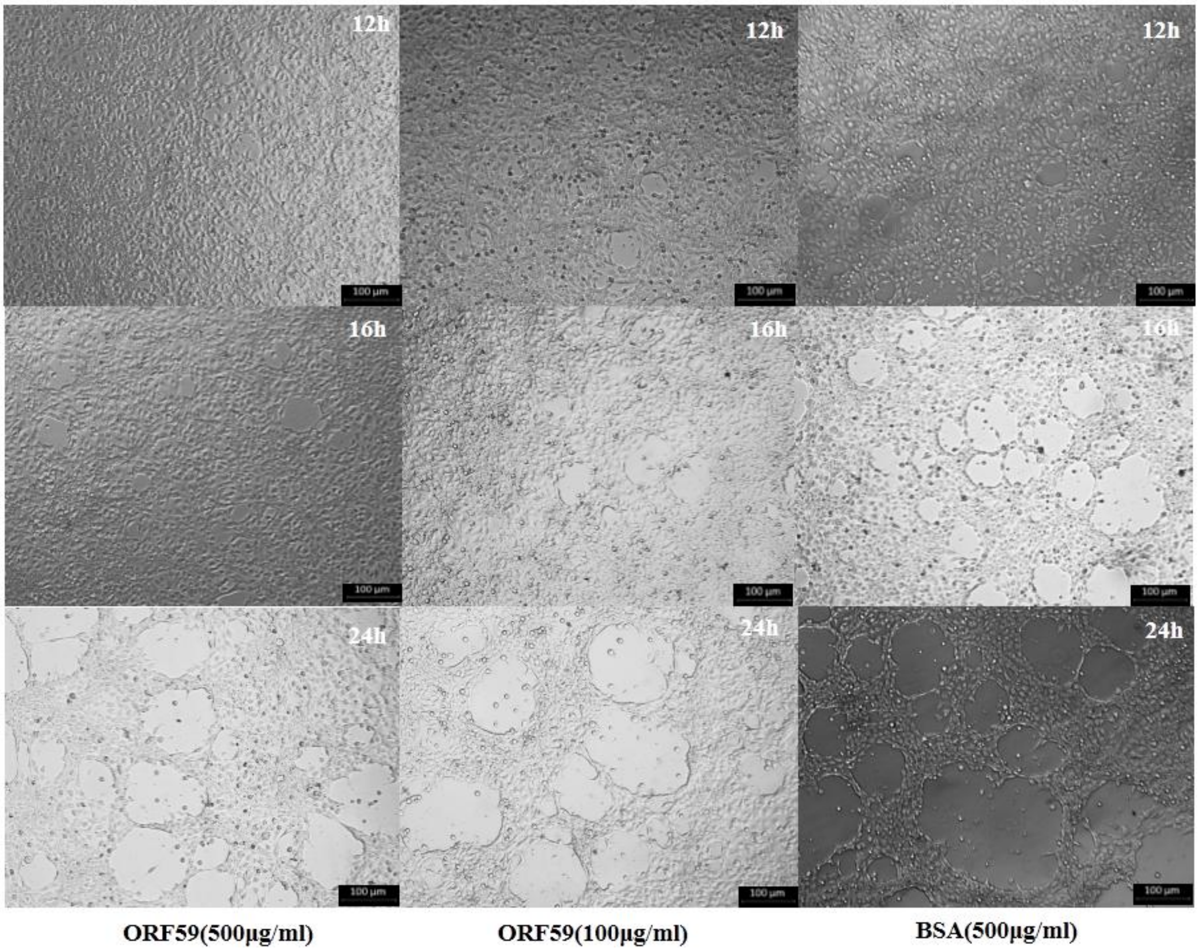

Figure. S2 Observation of Virus plaque. CCO cells were pre-incubated with different concentrations of ORF59 proteins (500 and 100 μg/ml) and BSA (500ug/ml) served as a negative control. The size of virus plaques was observed by microscope at 12, 16, and 24 hpi. Bar = 100 μm.

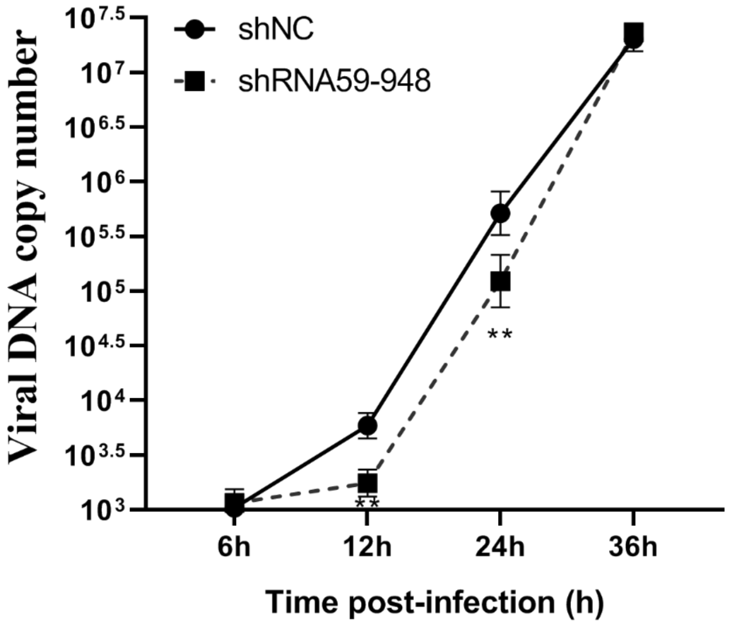

Figure. S3 Measurement of the copy number of CCV genomic DNA after ORF59 silencing. The progeny viruses within the cell supernatants at 36 hpi (shRNA59-948 and shRNA-NC) were collected to infect CCO cells, and the virus genome was measured using qPCR at 12, 16 and 24 hpi.
